# Supplementary material for: Impact of COVID-19 and effects of booster vaccination with BNT162b2 on six-month long COVID symptoms, quality of life, work productivity and activity impairment during Omicron
Source: J Patient Rep Outcomes. 2023 Jul 24;7:77. doi: 10.1186/s41687-023-00616-5 (PMC10366033; doi:10.1186/s41687-023-00616-5)
Supplement: Supplementary file 1 — Additional file 1: Figure S1 Study Flow Chart. Figure S2 Questionnaire on long COVID symptoms. Figure S3 Prevalence of long COVID symptoms by vaccination status. Figure S4 Number of symptoms over time by vaccination status. Figure S5 Absence of long COVID symptoms by vaccination status. [file 41687_2023_616_MOESM1_ESM.pptx]

## Slide 1
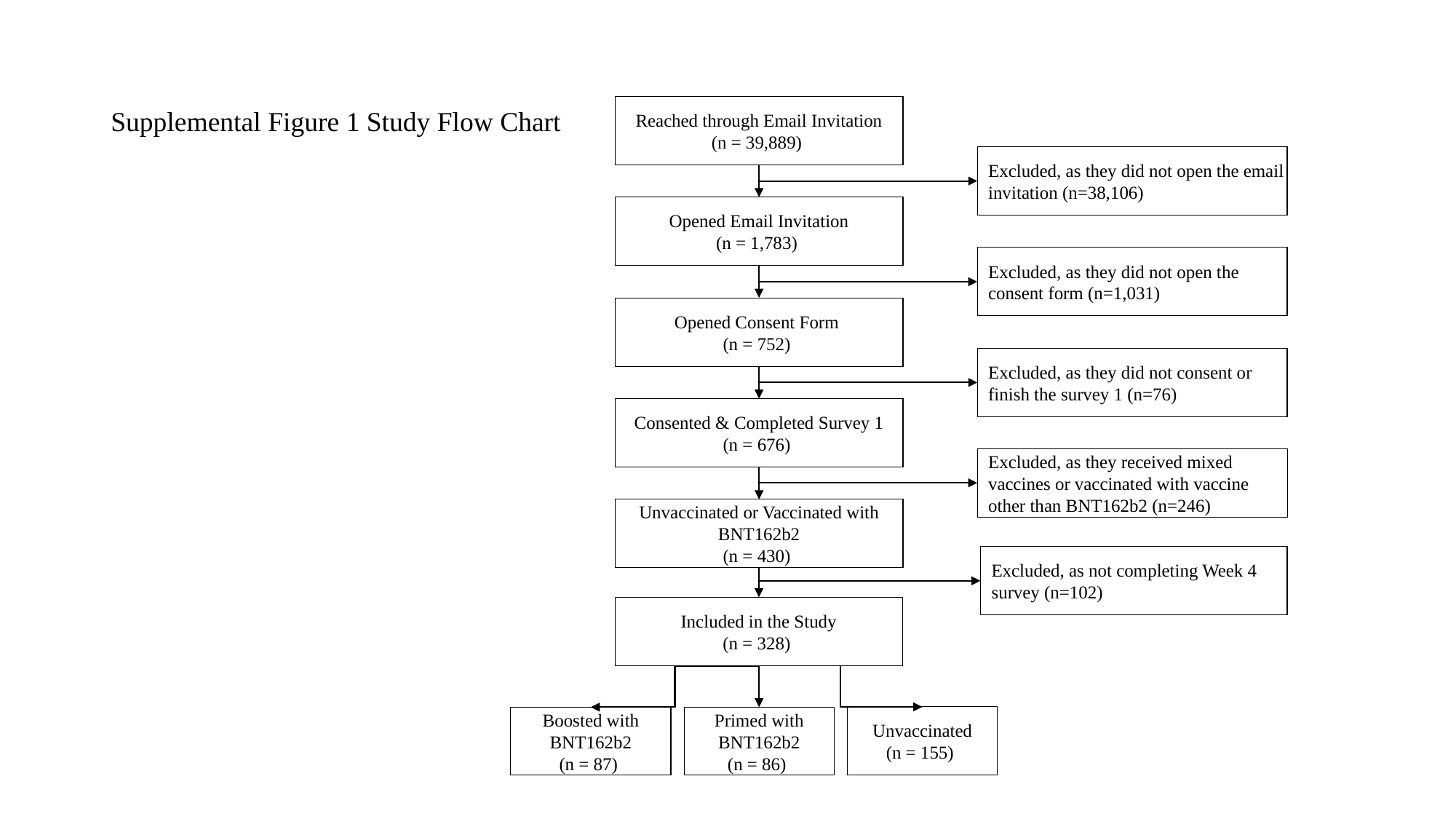

# Supplemental Figure 1 Study Flow Chart
Reached through Email Invitation
(n = 39,889)
Excluded, as they did not open the email invitation (n=38,106)
Opened Email Invitation
(n = 1,783)
Excluded, as they did not open the consent form (n=1,031)
Opened Consent Form
(n = 752)
Excluded, as they did not consent or finish the survey 1 (n=76)
Consented & Completed Survey 1
(n = 676)
Excluded, as they received mixed vaccines or vaccinated with vaccine other than BNT162b2 (n=246)
Unvaccinated or Vaccinated with BNT162b2
(n = 430)
Excluded, as not completing Week 4 survey (n=102)
Included in the Study
(n = 328)
Unvaccinated
(n = 155)
Boosted with BNT162b2
(n = 87)
Primed with BNT162b2
(n = 86)

## Slide 2
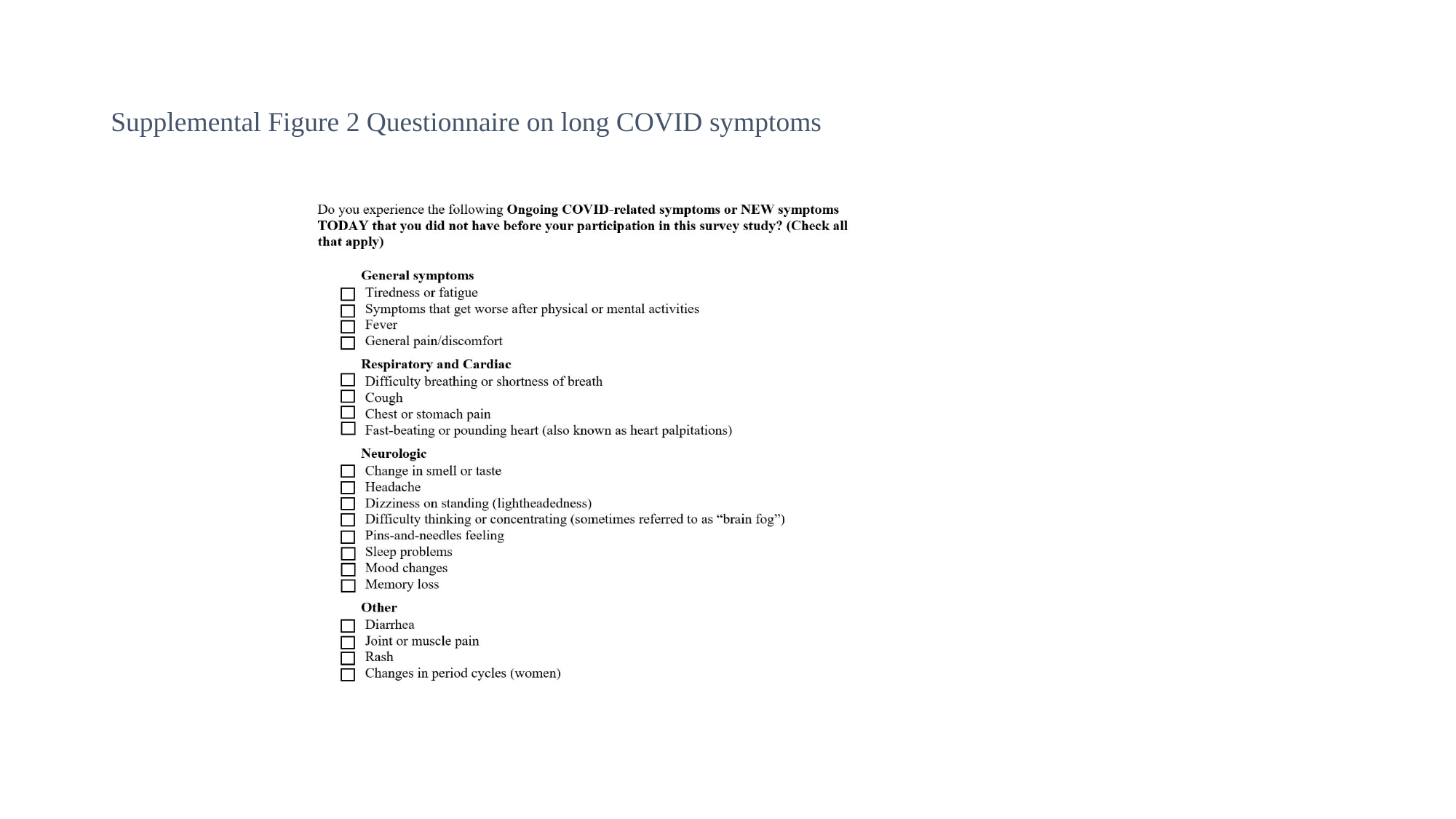

# Supplemental Figure 2 Questionnaire on long COVID symptoms

## Slide 3
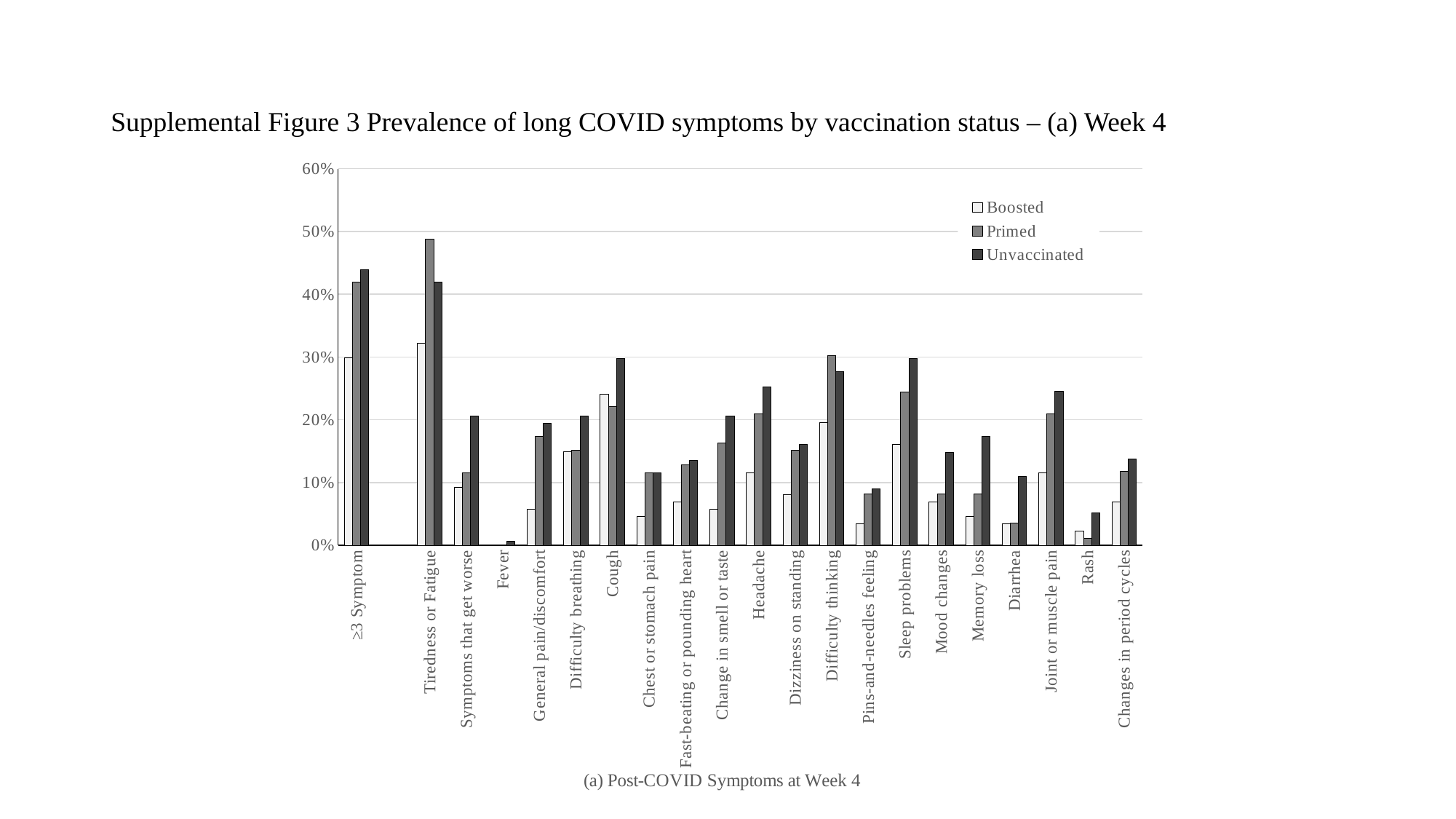

# Supplemental Figure 3 Prevalence of long COVID symptoms by vaccination status – (a) Week 4
### Chart
| Category | Boosted | Primed | Unvaccinated |
|---|---|---|---|
| ≥3 Symptom | 0.299 | 0.419 | 0.439 |
| | None | None | None |
| Tiredness or Fatigue | 0.322 | 0.488 | 0.419 |
| Symptoms that get worse | 0.092 | 0.116 | 0.206 |
| Fever | 0.0 | 0.0 | 0.0065 |
| General pain/discomfort | 0.0575 | 0.174 | 0.194 |
| Difficulty breathing | 0.149 | 0.151 | 0.206 |
| Cough | 0.241 | 0.221 | 0.297 |
| Chest or stomach pain | 0.046 | 0.116 | 0.116 |
| Fast-beating or pounding heart | 0.069 | 0.128 | 0.135 |
| Change in smell or taste | 0.0575 | 0.163 | 0.206 |
| Headache | 0.115 | 0.209 | 0.252 |
| Dizziness on standing | 0.0805 | 0.151 | 0.161 |
| Difficulty thinking | 0.195 | 0.302 | 0.277 |
| Pins-and-needles feeling | 0.0345 | 0.0814 | 0.0903 |
| Sleep problems | 0.161 | 0.244 | 0.297 |
| Mood changes | 0.069 | 0.0814 | 0.148 |
| Memory loss | 0.046 | 0.0814 | 0.174 |
| Diarrhea | 0.0345 | 0.0349 | 0.11 |
| Joint or muscle pain | 0.115 | 0.209 | 0.245 |
| Rash | 0.023 | 0.0116 | 0.0516 |
| Changes in period cycles | 0.069 | 0.118 | 0.138 |

## Slide 4
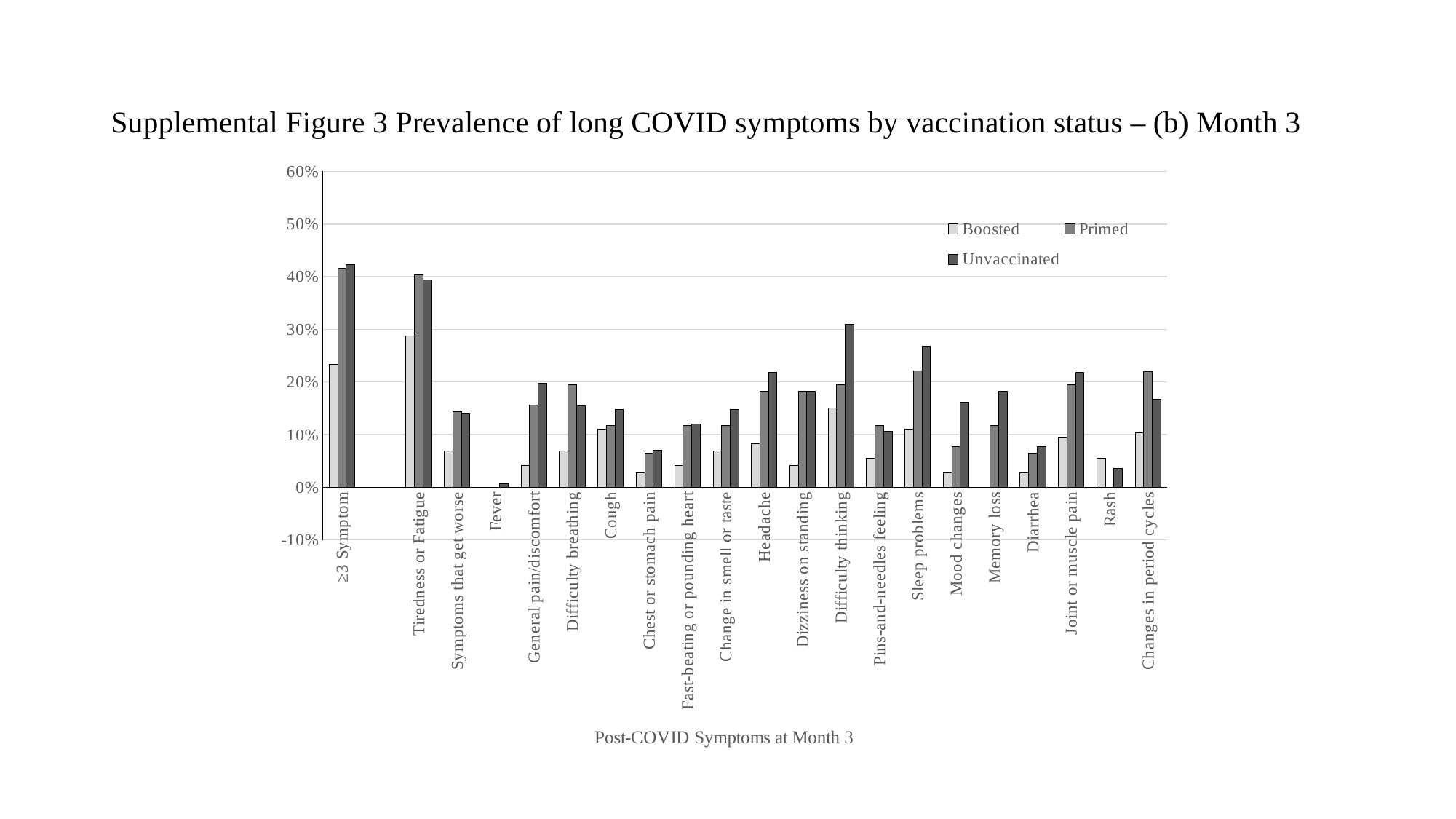

# Supplemental Figure 3 Prevalence of long COVID symptoms by vaccination status – (b) Month 3
### Chart
| Category | Boosted | Primed | Unvaccinated |
|---|---|---|---|
| ≥3 Symptom | 0.233 | 0.416 | 0.423 |
| | None | None | None |
| Tiredness or Fatigue | 0.288 | 0.403 | 0.394 |
| Symptoms that get worse | 0.0685 | 0.143 | 0.141 |
| Fever | 0.0 | 0.0 | 0.007 |
| General pain/discomfort | 0.0411 | 0.156 | 0.197 |
| Difficulty breathing | 0.0685 | 0.195 | 0.155 |
| Cough | 0.11 | 0.117 | 0.148 |
| Chest or stomach pain | 0.0274 | 0.0649 | 0.0704 |
| Fast-beating or pounding heart | 0.0411 | 0.117 | 0.12 |
| Change in smell or taste | 0.0685 | 0.117 | 0.148 |
| Headache | 0.0822 | 0.182 | 0.218 |
| Dizziness on standing | 0.0411 | 0.182 | 0.183 |
| Difficulty thinking | 0.151 | 0.195 | 0.31 |
| Pins-and-needles feeling | 0.0548 | 0.117 | 0.106 |
| Sleep problems | 0.11 | 0.221 | 0.268 |
| Mood changes | 0.0274 | 0.0779 | 0.162 |
| Memory loss | 0.0 | 0.117 | 0.183 |
| Diarrhea | 0.0274 | 0.0649 | 0.0775 |
| Joint or muscle pain | 0.0959 | 0.195 | 0.218 |
| Rash | 0.0548 | 0.0 | 0.0352 |
| Changes in period cycles | 0.104 | 0.22 | 0.167 |

## Slide 5
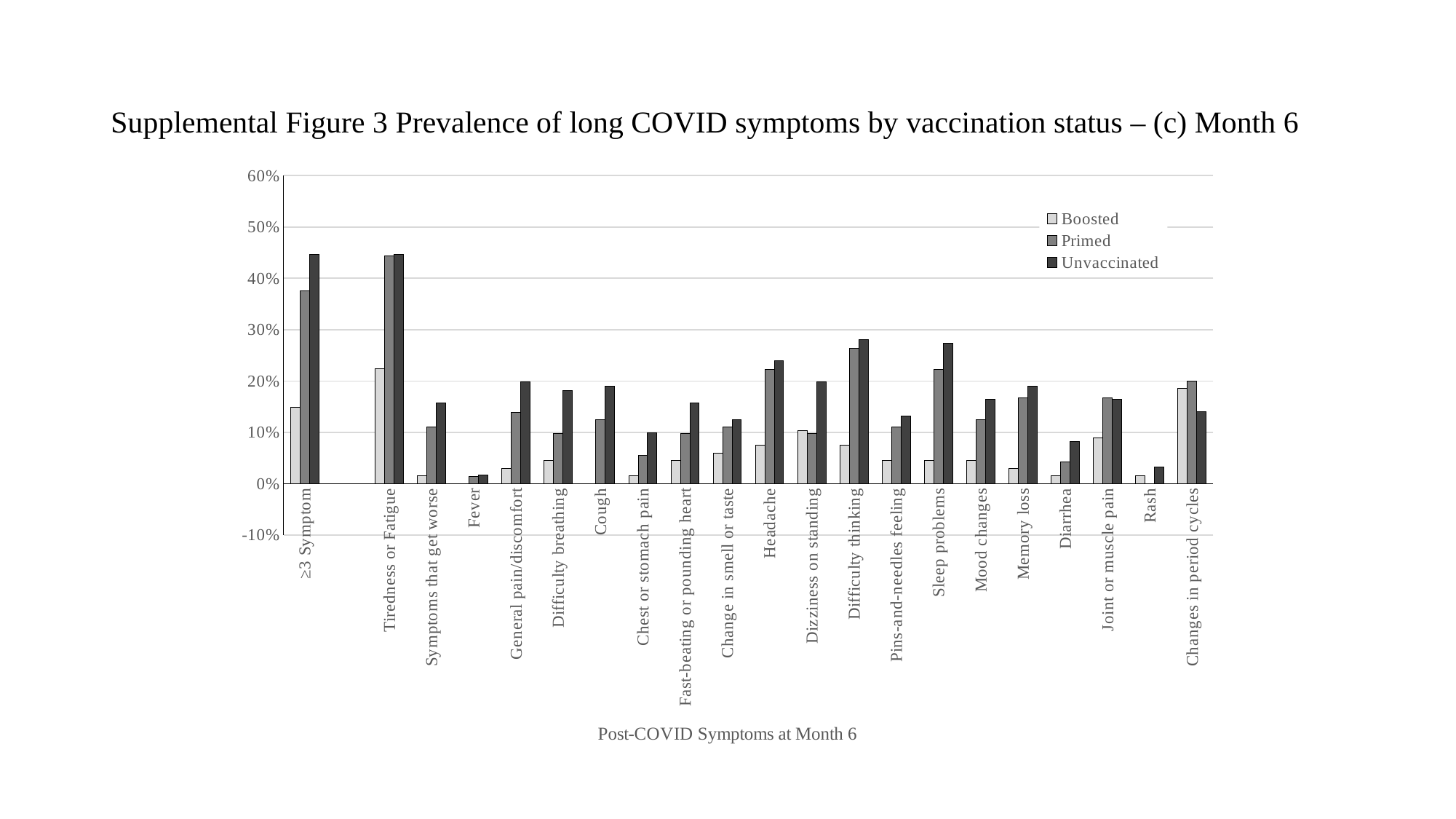

# Supplemental Figure 3 Prevalence of long COVID symptoms by vaccination status – (c) Month 6
### Chart
| Category | Boosted | Primed | Unvaccinated |
|---|---|---|---|
| ≥3 Symptom | 0.149 | 0.375 | 0.446 |
| | None | None | None |
| Tiredness or Fatigue | 0.224 | 0.444 | 0.446 |
| Symptoms that get worse | 0.0149 | 0.111 | 0.157 |
| Fever | 0.0 | 0.0139 | 0.0165 |
| General pain/discomfort | 0.0299 | 0.139 | 0.198 |
| Difficulty breathing | 0.0448 | 0.0972 | 0.182 |
| Cough | 0.0 | 0.125 | 0.19 |
| Chest or stomach pain | 0.0149 | 0.0556 | 0.0992 |
| Fast-beating or pounding heart | 0.0448 | 0.0972 | 0.157 |
| Change in smell or taste | 0.0597 | 0.111 | 0.124 |
| Headache | 0.0746 | 0.222 | 0.24 |
| Dizziness on standing | 0.104 | 0.0972 | 0.198 |
| Difficulty thinking | 0.0746 | 0.264 | 0.281 |
| Pins-and-needles feeling | 0.0448 | 0.111 | 0.132 |
| Sleep problems | 0.0448 | 0.222 | 0.273 |
| Mood changes | 0.0448 | 0.125 | 0.165 |
| Memory loss | 0.0299 | 0.167 | 0.19 |
| Diarrhea | 0.0149 | 0.0417 | 0.0826 |
| Joint or muscle pain | 0.0896 | 0.167 | 0.165 |
| Rash | 0.0149 | 0.0 | 0.0331 |
| Changes in period cycles | 0.186 | 0.2 | 0.14 |

## Slide 6
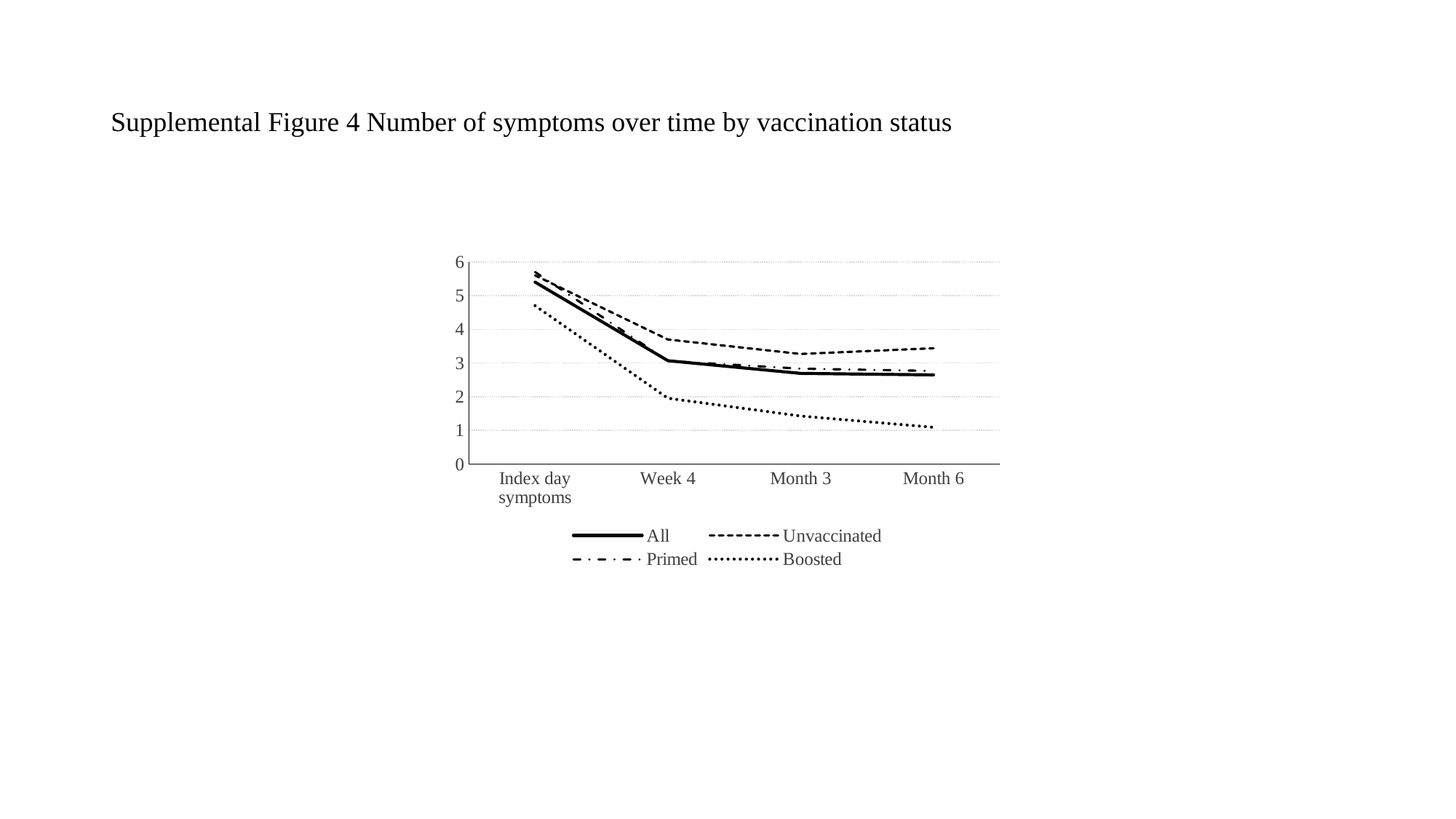

# Supplemental Figure 4 Number of symptoms over time by vaccination status
### Chart
| Category | All | Unvaccinated | Primed | Boosted |
|---|---|---|---|---|
| Index day symptoms | 5.4 | 5.6 | 5.7 | 4.7 |
| Week 4 | 3.067073170731707 | 3.696774193548387 | 3.058139534883721 | 1.9540229885057472 |
| Month 3 | 2.691780821917808 | 3.267605633802817 | 2.831168831168831 | 1.4246575342465753 |
| Month 6 | 2.646153846153846 | 3.43801652892562 | 2.763888888888889 | 1.0895522388059702 |

## Slide 7
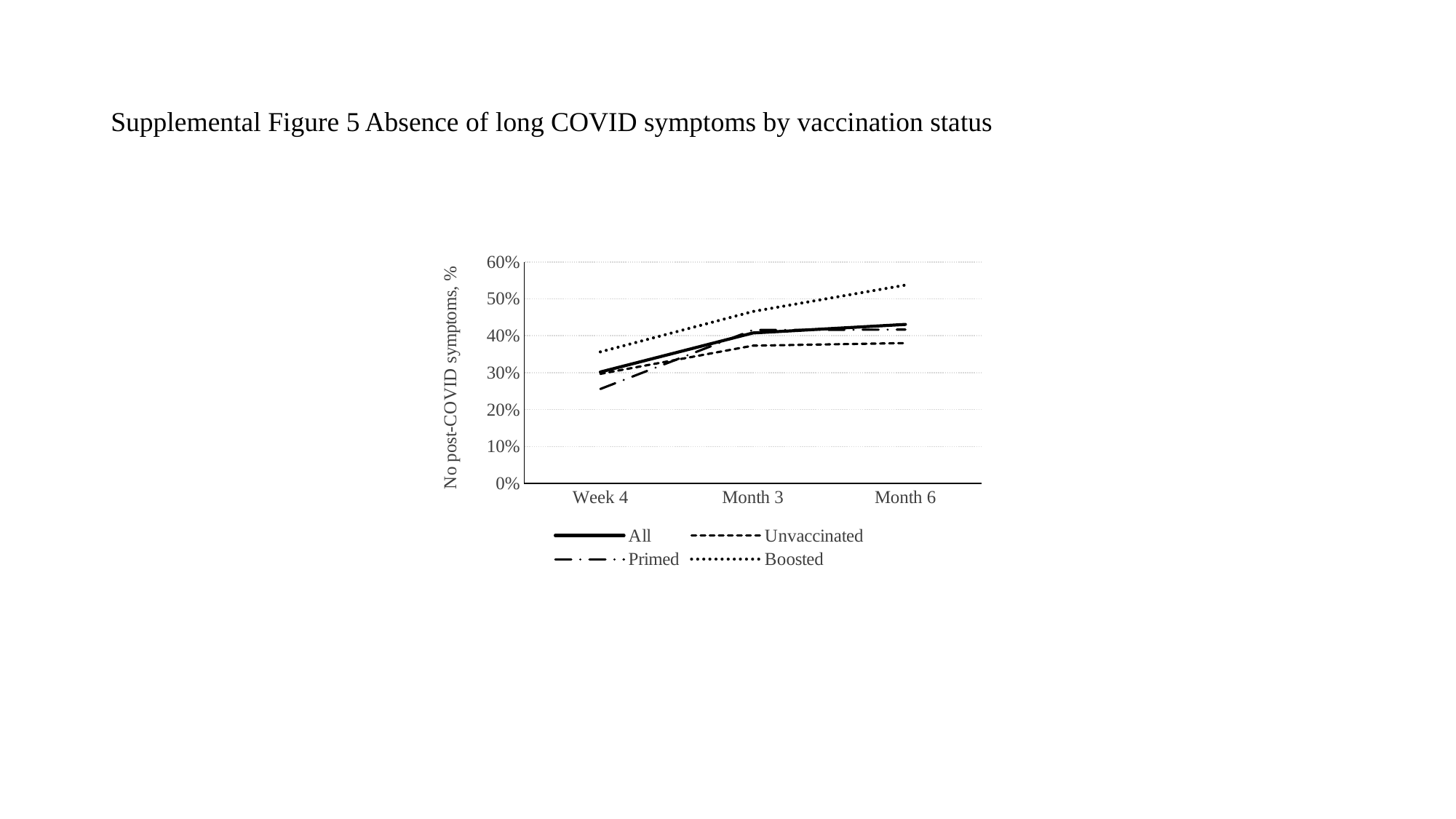

# Supplemental Figure 5 Absence of long COVID symptoms by vaccination status
### Chart
| Category | All | Unvaccinated | Primed | Boosted |
|---|---|---|---|---|
| Week 4 | 0.301829268292683 | 0.2967741935483871 | 0.2558139534883721 | 0.3563218390804598 |
| Month 3 | 0.40753424657534243 | 0.37323943661971826 | 0.4155844155844156 | 0.4657534246575342 |
| Month 6 | 0.4307692307692308 | 0.3801652892561983 | 0.41666666666666663 | 0.5373134328358209 |
